# Fluorescent tetracycline bone labeling as an intraoperative tool to debride necrotic bone during septic hip revision: a preliminary case series

**DOI:** 10.5194/jbji-6-85-2021

**Published:** 2021-01-27

**Authors:** Ernesto Muñoz-Mahamud, Jenaro Ángel Fernández-Valencia, Andreu Combalia, Laura Morata, Álex Soriano

**Affiliations:** 1 Department of Orthopaedics and Trauma Surgery, Hospital Clínic de Barcelona, University of Barcelona, Barcelona, Spain; 2 Department of Infectious Diseases, Hospital Clínic de Barcelona, University of Barcelona, Barcelona, Spain

## Abstract

A plausible cause of persistent infection after septic hip revision may be the presence of nonviable osteomyelitic bone. Since surgical excision of these necrotic fragments is often challenging, the use of fluorescent tetracycline bone labeling (FTBL) as an intraoperative tool may pose an additional assessment aid to provide a visual index of surgical debridement. **Methods**: We present a single-center study performed in a university hospital from January 2018 to June 2020, in which all consecutive cases of chronic hip periprosthetic joint infection (PJI) undergoing revision using FTBL were retrospectively reviewed. In all cases, the patient was under treatment with tetracyclines at the moment of the revision surgery. During the surgery, all bone failing to fluoresce was considered nonviable and thus removed and sent for both culture and histology. **Results**: We include three cases in which the FTBL technique was used. In all cases, the histopathological examinations of the nonfluorescent removed bone were consistent with chronic osteomyelitis. **Conclusion**: The intraoperative use of FTBL successfully aided the surgeon to detect the presence of nonviable bone in all the presented cases of chronic prosthetic hip infection.

## 1 Introduction

Failure rates after hip revision due to chronic periprosthetic joint infection (PJI) range from to 2.5 % to 15 % according to the literature (Hoberg et al., 2016). A highly plausible cause of persistent infection may be the presence of nonviable tissue, including necrotic fragments of bone. Despite bone samples being more difficult to grind, its obtention should not be disregarded in chronic PJI as reported yields range from 77 % to 78 % (Bémer et al., 2016). However, surgical excision of these necrotic fragments is often problematic, owing to the difficulty in distinguishing unviable bone from the surrounding live bone. For this concrete purpose, not many tools aid the surgeon during the procedure. Bone appearance is somehow subjective, and which pieces to excise depend on the surgeon's own judgment. Alternative techniques have been proposed to allow for visualization of chronically infected tissues, i.e., laser Doppler flowmetry, bone-autofluorescence detection or the use of intra-articular methylene blue (Duwelius and Schmidt, 1992; Pautke et al., 2010; Shaw et al., 2017; Swiontkowski, 1990). Notwithstanding, additional studies are still needed to elucidate their real usefulness, particularly in reference to cases of hip PJI.

For this reason, the use of fluorescent tetracycline bone labeling (FTBL) as an intraoperative tool has been proposed by some authors as an additional assessment aid to provide a visual index of surgical debridement. So far, most of the reported experience is related to the maxillofacial surgery field, where there is wide experience in treating medication-related osteonecrosis of the jaw (Dahners and Bos, 2002; Giudice et al., 2018; Harvey et al., 2004; Pautke et al., 2010, 2006; Yoshiga et al., 2015a). In this brief report, we present our experience in hip revision for chronic PJI in which FTBL was used.

## 2 Methods

We present a single-center study performed in a university hospital from January 2018 to June 2020, in which all consecutive cases of chronic hip PJI undergoing revision using FTBL were retrospectively reviewed. In all cases, the patient had been under a variable duration treatment with tetracyclines (minocycline 100 mg every 12 h orally) at the moment of the revision surgery. All cases were followed up for at least 12 months after the surgical intervention.

All operations were performed by hip infection surgeons (Ernesto Muñoz-Mahamud, Jenaro Ángel Fernández-Valencia and Andreu Combalia) in a laminar-airflow-fitted operating room. Multiple culture samples were obtained from each case, including synovial fluid as well as periprosthetic tissue samples. Once extensive debridement followed by high-pressure pulsatile lavage had been performed, a Wood's lamp (Escolite 51 LED wavelength 395 nm, Houston, TX, USA) was held over the surgical field in order to illuminate the remaining exposed bone, providing a manifest contrast between the viable and the necrotic bone under the ultraviolet light spectrum. All bone failing to fluoresce was considered nonviable and thus removed until fluorescent greenish bone appeared. Bright, glowing bone secondary to tetracycline uptake was considered viable irrigated bone. All removed nonviable bone was sent for both culture and histology.

The histology was considered positive for infection when ≥ 5 neutrophils per high-power field (400×) were found in at least five separate microscopic fields. Chronic osteomyelitis was defined as the microscopic presence of late fibrosis of marrow with chronic inflammatory infiltrate and plasma cell predominance along with fragments of necrotic bone and multinucleated giant cells.

## 3 Results

We present herein three cases of complex chronic PJI in which, after multiple surgical interventions, the latter revision was performed using the FTBL technique. The main characteristics of the cohort are summarized in Table 1.

**Table 1:** Main characteristics regarding the three cases of chronic PJI in which, after multiple surgical interventions, the latter revision was performed using the FTBL technique.

Case number	Age (years)	Sex	Femoral bone loss${}^{*}$	Causative microorganism	Tetracycline dosage and administrationperiod	Surgical intervention	Histological findings	Follow-up (12 months)
Case 1	67	Female	IIIA	Meticillin-resistant *Staphylococcus aureus*	Minocycline 100 mg every 12 h orallyfor 4 weeks	Spacer removal andreimplantation	Late fibrosis of marrow with chronic inflammatory infiltrate and plasma cell predominance along with fragments of necrotic bone, consistent with osteomyelitis; < 5 neutrophils per high-power field.	Absence of relapse
Case 2	40	Male	II	Meticillin-resistant *Staphylococcus aureus*	Minocycline 100 mg every 12 h orallyfor 10 d	First stage of a two-stage septic revision	Late fibrosis of marrow with chronic inflammatory infiltrate along with fragments of necrotic bone and bone marrow edema, consistent with osteomyelitis; > 5 neutrophils per high-power field.	Absence of relapse,waiting for the secondstage of the revision
Case 3	68	Female	I	*Escherichia coli*	Minocycline 100 mg every 12 h orally for 7 d	Reimplantation	Bone tissue with reactive vascular proliferation and chronic inflammatory infiltrate along with fragments of necrotic bone and bone marrow edema, consistent with osteomyelitis; < 5 neutrophils per high-power field.	Absence of relapse

${}^{*}$ Femoral bone loss according to the Paprosky classification (Weeden and Paprosky, 2002).

### 3.1 Case 1

A 67-year-old woman with diabetes mellitus, who had previously been operated on 13 times for chronic hip PJI, was referred to our hospital with a hip spacer and a sinus tract under the diagnostic of chronic PJI caused by meticillin-resistant *Staphylococcus aureus*. We initially performed extensive debridement and spacer exchange and then two additional spacer revisions. Finally, minocycline 100 mg every 12 h orally was administered for 4 weeks. Later, spacer removal and a hip megaprosthesis was implanted. During the surgery, all bone failing to fluoresce was removed and analyzed (Fig. 2). Bone cultures were all negative, whereas the histology depicted an elevated polymorphonuclear (PMN) count and informed about chronic osteomyelitis. After 14 months of follow-up, the patient is free of symptoms, all lab biomarkers remain unaltered and the patient is able to walk with one crutch.

**Figure 1:**
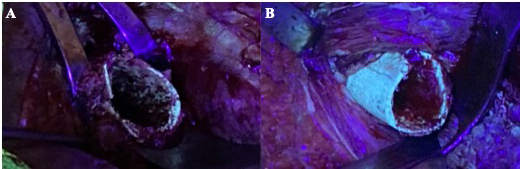
Intraoperative photographs of the surgical field using an ultraviolet lamp of a patient that had been receiving minocycline 100 mg every 12 h orally for 4 weeks (case 1). **(a)** Remaining bone showed almost no fluorescence under black light and was considered nonviable. **(b)** After resection of the necrotic bone, viable greenish glowing bone secondary to tetracycline uptake appeared.

**Figure 2:**
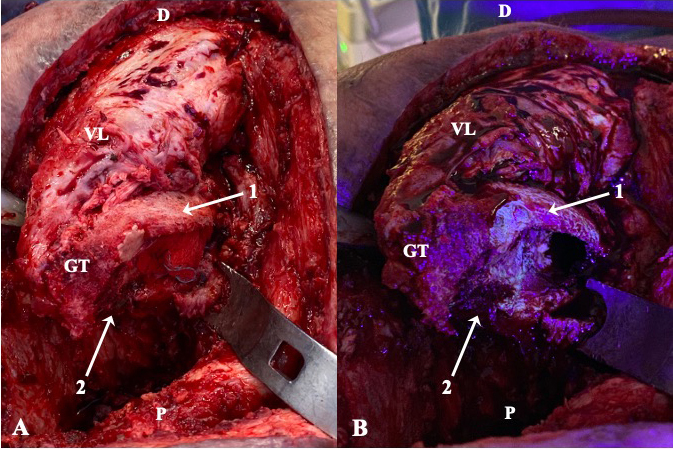
Intraoperative clinical photographs showing the ventral aspect of the proximal femur of a patient operated through anterolateral approach, who had been receiving minocycline 100 mg every 12 h orally for 10 d (case 2). The surgical field prior **(a)** and after **(b)** the utilization of fluorescent light is compared. Under the black light, viable metaphyseal bone glowed greenish (1), whereas all that bone failing to fluoresce (2) was considered necrotic and thus removed. D: distal; P: proximal; GT: greater trochanter; VL: vastus lateralis.

### 3.2 Case 2

A 40-year-old man was referred from another hospital for chronic PJI with a sinus tract caused by meticillin-resistant *Staphylococcus aureus.* The patient was a smoker, HVC (hepatitis C virus) positive and presented extreme obesity with a body mass index of 44.1 kg/m${}^{2}$. Two-stage revision was performed but reinfection occurred. Two additional debridement procedures were performed, in which *Enterobacter cloacae* AMPc (cyclic adenosine 3${}^{\prime }$,5${}^{\prime }$-monophosphate) was isolated. Extended-spectrum antibiotic treatment was initialized, including minocycline 100 mg every 12 h orally, which was administered for 10 d prior to the latter revision. During this surgery, some bone areas failed to fluoresce so they were removed and analyzed. Histology from bone samples showed elevated PMN count as well as chronic osteomyelitis. After 12 months of follow-up, the patient is free of symptoms regarding the hip spacer and able to walk short distances with two crutches. He is being multidisciplinary managed under an extreme diet and does not accept further surgeries until significant weight loss is achieved.

### 3.3 Case 3

A 68-year-old woman with a seropositive polyarthritis presented in the emergency room with a 3 d history of acute hip pain and fever. The patient was diagnosed of acute hip septic arthritis and was immediately debrided. During the intervention, significant destruction of the cartilage was depicted, and a hip spacer was implanted. All cultures were positive for *Escherichia coli*. Antibiogram-based antibiotics were initialized, yet the evolution of the patient was unfair, so she needed two additional debridement surgeries. In the last one, no spacer was implanted. Finally, the second stage was performed with the patient under oral antibiotic treatment including minocycline 100 mg every 12 h orally, started 1 week prior. During the surgery, those bone areas considered unviable under the black light were removed and sent for analysis. Bone cultures turned out to be negative, whereas the histology revealed osteomyelitic findings. After 14 months of follow-up, the patient is free of symptoms related to the hip and is able to walk without pain using two crutches.

## 4 Discussion

A plausible cause of persistent infection after septic hip revision may be the presence of nonviable osteomyelitic bone in the proximal part of the femur in contact with the articular cavity. In fact, it has been demonstrated that implant-associated osteomyelitis entails not only destruction of the implant cavity contour but also the presence of an avascular zone of necrotic bone tissue and hypoxia (Jensen et al., 2017). These local tissue findings may result in decreased penetration of antibiotics and insufficient oxygen supply. For this reason, bone sampling should be always ground and sent for both culture and histological analysis. However, surgical excision of those nonviable bone fragments is often challenging, owing to the elusiveness in distinguishing it from the surrounding live bone. For this reason, it seems an attractive idea to take advantage of the administration of tetracyclines to provide an intraoperative visual index of surgical debridement.

Tetracycline is a classic antibiotic and is well known to be taken up in newly formed bone in order to conform an area that is brightly fluorescent under black light (Perrin, 1965). Therefore, tetracycline is rapidly accumulated at the site of active bone remodeling by chelating with free calcium. This quick deposition in bone turns out to reflect wavelengths of light in the ultraviolet spectrum. However, there is currently no clear evidence regarding the optimal minimum period of tetracycline administration so as to obtain significant reliable fluorescence during a surgical procedure. Indeed, Harvey et al. (2004) reported that tetracycline administration 48 h prior to surgery may be enough to achieve fair bone uptake. On the contrary, Pautke et al. (2006) reported periods that range from days to “several weeks”, whereas Dahners and Bos (2002) recommend extended periods that range from 3 to 6 months in order to avoid faint labeling. Yoshiga et al. (2015b) reported a series in which at least 10 d of tetracycline administration seemed enough so as to differentiate between fluorescent and nonfluorescent areas. In our experience, the most glowing tetracycline uptake was achieved in that case in which the antibiotic had been administered for 4 weeks (Table 1).

It seems reasonable to think that the longer the tetracycline is administered, the brighter and more evident the uptake will be. Subjective intraoperative findings, for instance bone appearance or bleeding, may not be reliable enough and may not exactly correlate with the real bone viability, so additional intraoperative assistance may be recommended. It is important to remember that this technique is also subject to the surgeon's interpretation, so it might be only used as an aid for intraoperative decision-making, whereas the final decision to remove bone should be based on the overall bone study results including both preoperative and intraoperative tests.

It has been reported that tetracycline administration may be stopped a week before surgery without an effect on the labeling, in case that both bone-labeling and intraoperative cultures are needed (Dahners and Bos,, 2002). Furthermore, it should be taken into account that recent studies suggest that two-stage exchange with a short interval in which non-stopped active therapy is used is as effective as two-stage revision with longer intervals in which the antibiotic is discontinued (Winkler et al., 2019). Therefore, as in the present series, those cases in which the chosen active antibiotic is a tetracycline could take advantage of this intraoperative tool to detect the presence of nonviable bone.

We report herein a brief report regarding a short case series, in which the major limitation is related to the small size of the sample, which may affect the extent to which our findings can be generalized beyond the specific cases studied. In addition, because of its retrospective nature, certain biases might have influenced the results. However, much of the data analyzed and the final conclusion of the study are unlikely to be affected by this fact. Furthermore, the novel applicability of this technique in reference with the orthopedic infectious surgery field may be valuable for those who are regularly involved with complex chronic PJIs.

Indeed, this preliminary study may serve as a basis for larger studies, which should be undertaken in order to set a recommendation regarding the optimal minimum period of tetracycline administration to obtain significant reliable fluorescence during the surgery and to evaluate its real usefulness to diminish the failure rate after the second stage of a septic revision. On the other hand, since bone seems to show its own auto-fluorescence, further efforts should be focused on elucidating the real necessity of the tetracycline labeling to distinguish viable from nonviable bone under the ultraviolet light. In fact, a recent randomized controlled study suggested that auto-fluorescence-guided bone surgery has comparable success rates to the FTBL surgery (Ristow et al., 2017).

In all, we conclude that the intraoperative use of FTBL may be a helpful aid for the surgeon to detect the presence of nonviable bone in chronic hip infection.

## Supplement

10.5194/jbji-6-85-2021-supplement The supplement related to this article is available online at: https://doi.org/10.5194/jbji-6-85-2021-supplement.

## Data Availability

For access to raw data, contact the corresponding author (Ernesto Muñoz-Mahamud: emunoz@clinic.cat).

## Code availability

The codes for each of the included patient in the series can be found in the anonymized database in the Supplement.
