# Supplementary material for: Fluorescent tetracycline bone labeling as an intraoperative tool to debride necrotic bone during septic hip revision: a preliminary case series
Source: J Bone Jt Infect. 2021 Jan 27;6(4):85–90. doi: 10.5194/jbji-6-85-2021 (PMC8132458; doi:10.5194/jbji-6-85-2021)
Supplement: The supplement related to this article is available online at: https://doi.org/10.5194/jbji-6-85-2021-supplement. [file jbji-6-85-supplement.zip › jbji-6-85-2021-supplement-title-page.pdf]

Supplement of J. Bone Joint Infect., 6, 85–90, 2021
https://doi.org/10.5194/jbji-6-85-2021-supplement
© Author(s) 2021. This work is distributed under
the Creative Commons Attribution 4.0 License.

JBJI
Journal of Bone and Joint Infection
Open Access

Supplement of

Fluorescent tetracycline bone labeling as an intraoperative tool to debride necrotic bone during septic hip revision: a preliminary case series

Ernesto Muñoz-Mahamud et al.

Correspondence to: Ernesto Muñoz-Mahamud (emunoz@clinic.cat, e.munoz.mahamud@gmail.com)

- jbji-6-85-2021-supplement-title-page.pdf
- availability code.xlsx

The copyright of individual parts of the supplement might differ from the CC BY 4.0 License.
